# Precision Solar Spectrum Filtering in Aerogel Windows via Synergistic ITO-Ag Nanoparticle Doping for Hot-Climate Energy Efficiency

**DOI:** 10.3390/gels11070553

**Published:** 2025-07-18

**Authors:** Huilin Yang, Maoquan Huang, Mingyang Yang, Xuankai Zhang, Mu Du

**Affiliations:** 1 Institute for Advanced Technology, Shandong University, Jinan 250061, China; yhl_1229@163.com (H.Y.); hmq@mail.sdu.edu.cn (M.H.); 4118003156@sdu.edu.cn (X.Z.); 2 School of Resources Engineering, Xi’an University of Architecture and Technology, Xi’an 710049, China; 3 Shenzhen Research Institute of Shandong University, Shenzhen 518057, China; 4 Shandong Key Laboratory of Thermal Science and Smart Energy Systems, Shandong University, Jinan 250061, China

**Keywords:** silica aerogel, plasmonic particle, energy-saving window

## Abstract

Windows are a major contributor to energy loss in buildings, particularly in hot climates where solar radiation heat gain significantly increases cooling demand. An ideal energy-efficient window must maintain high visible light transmittance while effectively blocking ultraviolet and near-infrared radiation, presenting a significant challenge for material design. We propose a plasma silica aerogel window utilizing the local surface plasmon resonance effect of plasmonic nanoparticles. This design incorporates indium tin oxide (ITO) nanospheres (for broad-band UV/NIR blocking) and silver (Ag) nanocylinders (targeted blocking of the 0.78–0.9 μm NIR band) co-doped into the silica aerogel. This design achieves a visible light transmittance of 0.8, a haze value below 0.12, and a photothermal ratio of 0.91. Building simulations indicate that compared to traditional glass, this window can achieve annual energy savings of 20–40% and significantly reduce the economic losses associated with traditional glass, providing a feasible solution for sustainable buildings.

## 1. Introduction

Buildings are responsible for approximately 40% of global energy consumption, with conventional windows acting as thermal “holes” that contribute to nearly 60% of this building-related energy exchange [1,2,3,4]. In hot climates, excessive solar heat gain through windows is the primary driver of cooling loads, creating an urgent demand for advanced glazing technologies [5,6]. The ultimate goal is to create an ideal energy-saving window that possesses high spectral selectivity, enabling maximum transmission of visible light (0.38−0.78 μm) for daylighting while strictly blocking solar heat from the ultraviolet (UV, <0.38 μm) and near-infrared (NIR, >0.78 μm) regions. To reduce building energy consumption through window units, researchers have conducted studies focusing on glass surface coatings and window films. Yu et al. [7] proposed a five-layer submicron dielectric multilayer structure coated on glass surfaces, achieving a high visible light transmittance of 86%, but with limited near-infrared radiation blocking capability. Liu et al. [8] developed a transparent ultra-high-molecular-weight polyethylene composite film, which maintains a visible light transmittance of >70% while effectively blocking UV (>90%) and NIR (>70%). In addition to applying films and coatings on glass surfaces, enhancing the spectral selectivity of glass substrate materials also presents a viable approach. In recent years, silica aerogels have gained recognition as an ideal solution for enhancing building insulation across diverse climates due to their exceptional optical properties and low thermal conductivity, demonstrating significant potential when integrated with architectural glass [9,10]. While pure silica aerogels have emerged as super-insulating materials, their relatively high manufacturing costs and inherent optical properties fall short of achieving this demanding spectral control [11,12,13].

To impart spectral selectivity to aerogels, doping with functional metal nanoparticles has become the most promising strategy [14,15,16]. This approach leverages phenomena like localized surface plasmon resonance (LSPR) to tailor radiative properties. The LSPR effect refers to a localized electromagnetic resonance phenomenon that occurs when free electrons on the surface of metal nanoparticles are excited by incident light. Metal nanoparticles exhibiting LSPR demonstrate strong photon absorption characteristics. For instance, indium tin oxide (ITO) nanoparticles are known to absorb UV and parts of the NIR spectrum while maintaining visible transparency [17]. However, a critical performance gap exists: ITO’s absorption efficiency significantly weakens in the lower NIR range (0.78−0.9 μm), a region that still carries substantial solar energy. On the other hand, plasmonic nanoparticles like silver (Ag) or gold (Au) can exhibit intense, tunable absorption peaks via LSPR [18,19,20]. Yet, a single type of plasmonic particle offers only narrowband absorption, failing to provide the comprehensive NIR shielding required. A seemingly straightforward solution—co-doping with multiple particle types—unveils a fundamental dilemma: achieving broad spectral coverage often requires high particle concentrations. This inevitably leads to severe parasitic light scattering, which dramatically increases haze and degrades optical clarity and can even compromise the aerogel’s delicate nanostructure, thereby increasing its thermal conductivity. This trade-off between broadband optical selectivity, visual transparency, and thermal insulation represents the key bottleneck hindering the development of high-performance aerogel windows.

This study directly confronts this challenge by proposing a synergistic plasmonic co-doping strategy to create a silica aerogel window with superior performance across all key metrics. Our innovation lies in the meticulous selection and combination of ITO nanospheres and Ag nanocylinders, where they act in concert rather than as a simple mixture. The ITO nanospheres provide a foundational, broadband blockade of UV and NIR radiation. Crucially, we then introduce geometrically tuned Ag nanocylinders as a “plasmonic scalpel” to precisely excise the remaining NIR radiation in the 0.78−0.9 μm window where ITO is inefficient. The LSPR effect enables this targeted absorption to be highly efficient even at extremely low doping concentrations. This low-concentration approach is the key to our breakthrough, allowing us to achieve comprehensive solar modulation while simultaneously preserving the aerogel’s intrinsic high visible transmittance, ultra-low haze, and excellent thermal insulation.

In this paper, we systematically investigate this concept. We first employ the T-matrix method and Monte Carlo simulations to model the radiative properties of the composite aerogel, optimizing particle dimensions, concentrations, and aspect ratios. The performance of the optimized design is then evaluated based on key metrics, including transmittance, haze, and the light-to-solar gain ratio. Finally, we conduct a comprehensive building energy consumption analysis to evaluate the tangible energy-saving potential of our plasmonic aerogel window. The results demonstrate that our synergistic doping strategy provides a robust and highly effective solution for next-generation energy-efficient glazing.

## 2. Results and Discussion

### 2.1. Model Validation

**Figure 1:**
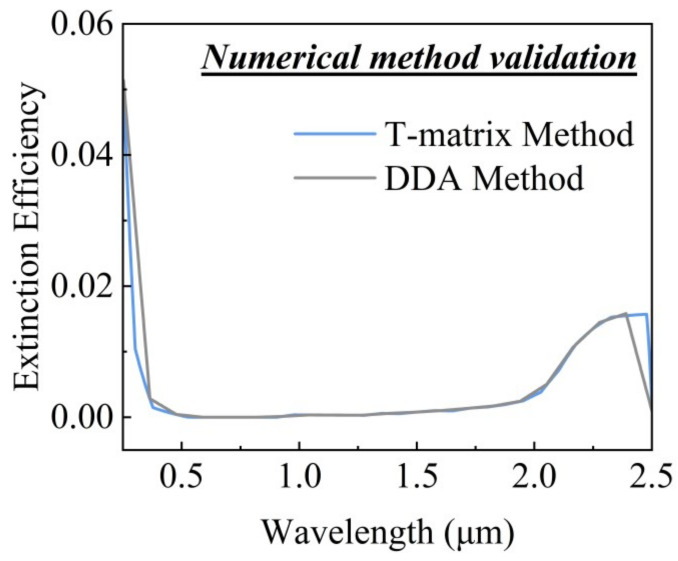
Extinction efficiency calculated by T-matrix and DDA methods.

To ensure the accuracy of our numerical simulations, the T-matrix code implemented in this study was validated. We calculated the extinction efficiency of ITO nanospheres with an effective radius of 5 nm using both the discrete dipole approximation (DDA) and T-matrix methods. As shown in Figure 1, the extinction efficiency obtained through the T-matrix method aligns closely with that from the DDA method, confirming the reliability of the T-matrix code in this work.

### 2.2. Engineering the Plasmonic Building Blocks for Spectral Control

To construct a window with ideal spectral selectivity, we first engineered the fundamental plasmonic “building blocks” capable of manipulating specific regions of the solar spectrum. Our strategy employs ITO nanospheres as a broadband filter and Ag nanocylinders as a tunable, precision tool to address ITO’s deficiencies.

**Figure 2:**
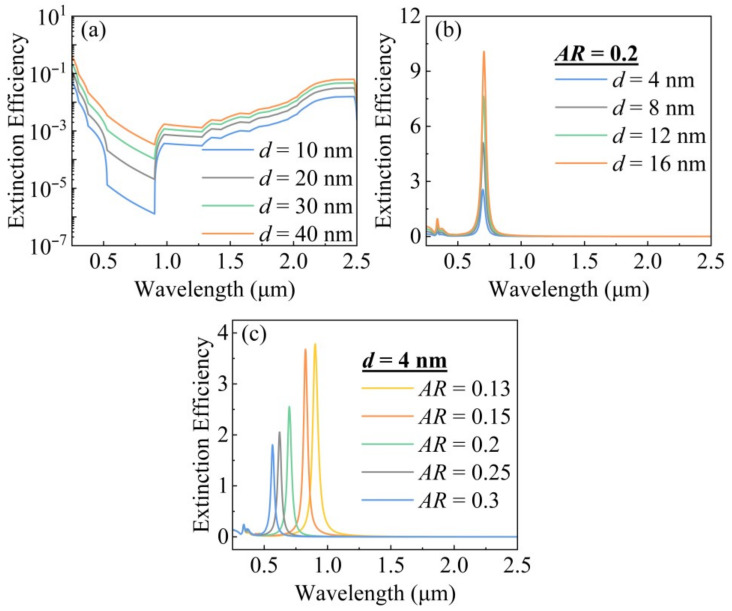
Extinction efficiency as a function of wavelength for (**a**) different diameters of ITO nanospheres, (**b**) different effective diameters of Ag cylinders, and (**c**) different aspect ratios of Ag nanocylinders.

Figure 2a illustrates that smaller ITO nanospheres exhibit reduced parasitic extinction in the visible range, improving transparency, while maintaining effective extinction in the UV and higher NIR regions (>1.2 μm). However, ITO’s weakness lies in its poor extinction between 0.78 μm and 1.2 μm, creating a spectral window for unwanted solar heat gain in the lower NIR range.

To precisely target this spectral gap, we introduced Ag nanocylinders, whose LSPR absorption peak can be programmed by tuning their geometry. Figure 2b demonstrates that although increasing the diameter of Ag nanocylinders enhances the extinction effect, it has a negligible effect on the position of the LSPR peak. A key tuning mechanism is revealed in Figure 2c. At a fixed diameter (*d* = 4 nm), decreasing the aspect ratio (*AR*) of the Ag nanocylinders induces a significant red shift of the extinction peak. It demonstrates that we can precisely set the absorption peak of the Ag nanocylinders to fill the absorption gap of ITO. For instance, nanocylinders with *AR* = 0.15 and *AR* = 0.13 exhibit sharp extinction peaks centered around 0.8 μm and 0.9 μm, respectively—perfectly aligning with our target. This “programmable” absorption provides the physical foundation for our synergistic doping strategy.

### 2.3. Synergistic Doping: Achieving Superior Spectral Selectivity

**Figure 3:**
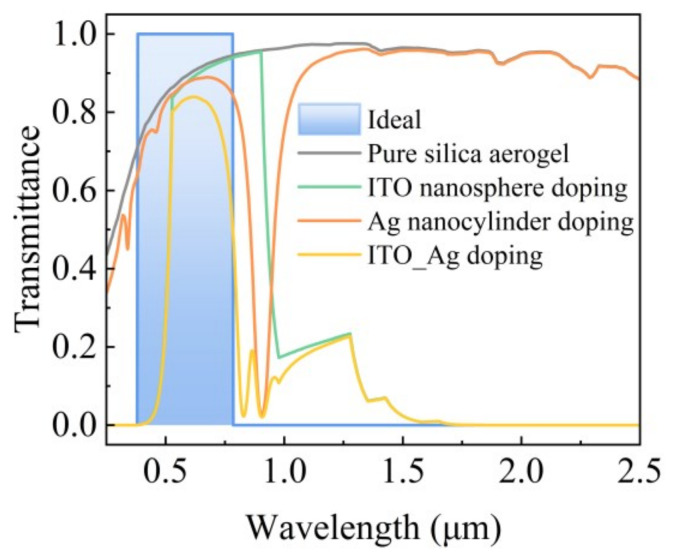
Solar transmittance of 2.5 mm aerogel doped with different nanoparticles.

To visualize the impact of doped nanoparticles on the aerogel’s radiation characteristics, we compared the solar transmittance of pure silica aerogel with that of aerogels doped with ITO nanospheres, Ag nanocylinders, and combinations of both. All samples had a thickness of 2.5 mm. Figure 3 shows that adding ITO and Ag nanoparticles significantly increases the selective transmission of solar radiation, enhancing UV and NIR absorption. This achieves high transmittance in the visible spectrum while effectively blocking both UV and NIR radiation.

### 2.4. Optimization Towards Peak Performance

**Figure 4:**
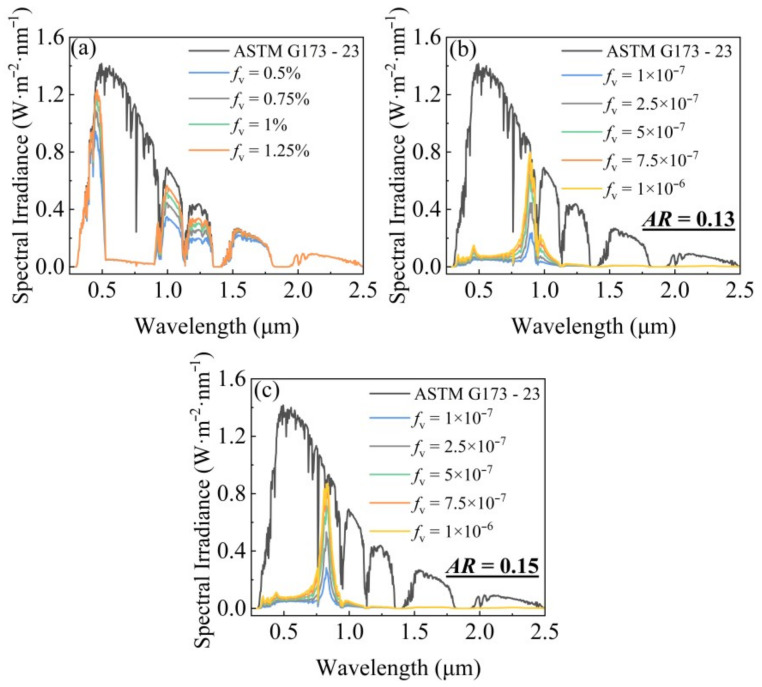
Solar absorption properties of (**a**) ITO nanospheres, (**b**) Ag nanocylinders with an aspect ratio of 0.13, and (**c**) Ag nanocylinders with an aspect ratio of 0.15 doped aerogels at varying doping concentrations.

Figure 4a–c illustrate the results for aerogels where ITO nanospheres with a diameter of 10 nm are doped at concentrations ranging from 0.5% to 1.25%, while Ag nanocylinders with an equivalent diameter of 4 nm are doped at concentrations from 1 × 10^−7^ to 1 × 10^−6^, considering aspect ratios of 0.13 and 0.15. Figure 4a demonstrates that increasing the concentration of ITO nanospheres leads to enhanced absorption of both UV and NIR light, with a relatively minor impact on visible light absorption within this concentration range. However, the absorption in the NIR band of 0.78–0.9 µm remains suboptimal with ITO alone. Figure 4b,c show that even low concentrations of Ag nanocylinders significantly boost NIR absorption. The optimal absorption peaks for Ag nanocylinders are dependent on their aspect ratio, with an aspect ratio of 0.13 showing a peak around 0.9 µm and an aspect ratio of 0.15 showing a peak around 0.8 µm. This confirms that by carefully selecting the aspect ratios and concentrations of Ag nanocylinders, the NIR absorption can be precisely tuned.

To further optimize the spectral performance, the combined effect of ITO nanospheres with a diameter of 10 nm and Ag nanocylinders with an equivalent diameter of 4 nm with two different aspect ratios (0.13 and 0.15) is numerically investigated. A “concentration ratio” is defined as the concentration of Ag nanocylinders with an aspect ratio of 0.13 relative to those with an aspect ratio of 0.15 (details in Table 1). The doping concentration for Ag nanocylinders with an aspect ratio of 0.13 is fixed at 1 × 10^−6^, corresponding to its absorption peak around 0.9 µm. The study then examines the solar transmittance of these spectrally selective aerogels at various thicknesses ranging from 2.5 mm to 3.5 mm, with ITO nanosphere concentrations ranging from 0.5% to 1.25% and Ag nanocylinder concentration ratios from 1 to 10.

**Figure 5:**
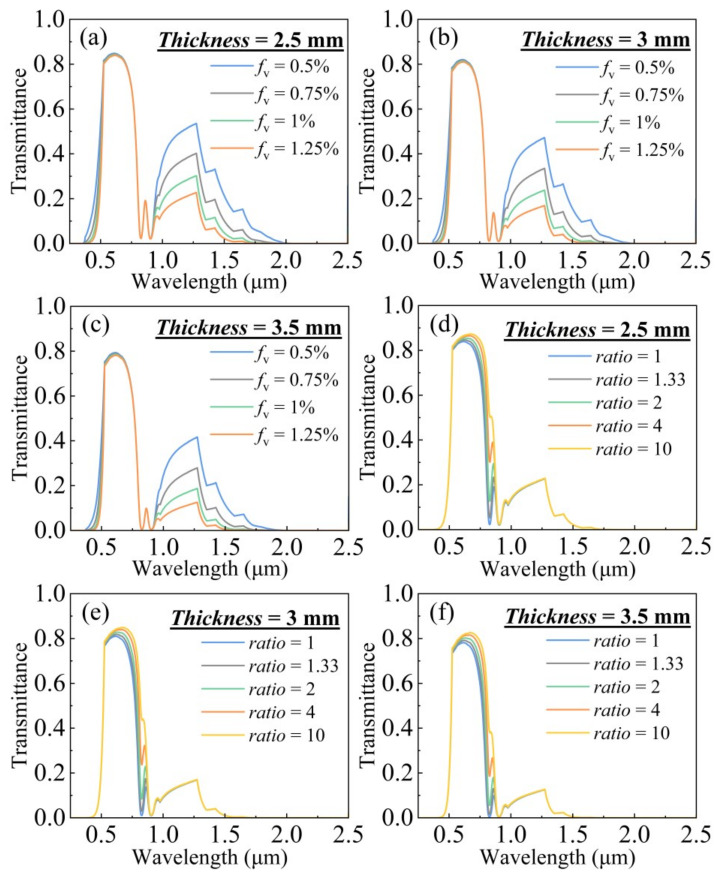
Effect of ITO nanosphere doping concentration on transmittance of aerogel glass with different thicknesses (**a**–**c**). Effect of Ag nanocylinder doping concentration ratio on transmittance of aerogel glass with different thicknesses (**d**–**f**).

Figure 5a–c depict the effect of varying ITO nanosphere doping concentration on the overall transmittance for aerogel thicknesses from 2.5 mm to 3.5 mm. As the ITO nanosphere concentration increases, NIR transmittance in the 1–2.5 µm range decreases significantly, accompanied by a reduction in UV transmittance. Concurrently, as the aerogel thickness increases, visible light transmittance experiences a slight decrease, and the maximum NIR transmittance drops from approximately 0.55 to 0.4. This behavior is attributed to the enhanced light absorption and scattering resulting from higher doping concentrations and greater optical path lengths in thicker materials. Figure 5d–f illustrate the impact of varying the doping concentration ratio of Ag nanocylinders on transmittance for thicknesses between 2.5 mm and 3.5 mm. A decrease in doping concentration ratio results in a significant reduction in NIR transmittance specifically within the 0.78–0.8 µm range. Again, increased thickness leads to a slight decline in overall transmittance due to heightened absorption and scattering. The strategic combination of ITO nanospheres and appropriately tuned Ag nanocylinders within the silica aerogel substrate clearly demonstrates the ability to augment absorption in the UV and broad NIR regions while striving to maintain high visible light transmission.

For practical applications in building energy efficiency and aesthetics, a comprehensive evaluation of the spectrally selective aerogel glass was conducted, analyzing the influence of aerogel thickness, ITO nanosphere doping concentration, and Ag nanocylinder doping concentration ratio on key performance metrics: solar transmittance (*T*_sol_), visible light transmittance (*T*_ρ_), human vision-based visible light transmittance (*T*_lum_), light-to-solar heat gain ratio (*LSG*), deviation from an ideal spectrum (*σ*_T_), and haze. The parameter ranges were thickness from 2.5 mm to 3.5 mm, ITO nanosphere concentration from 0.5% to 1.25%, and Ag nanocylinder concentration ratio from 1 to 10.

**Figure 6:**
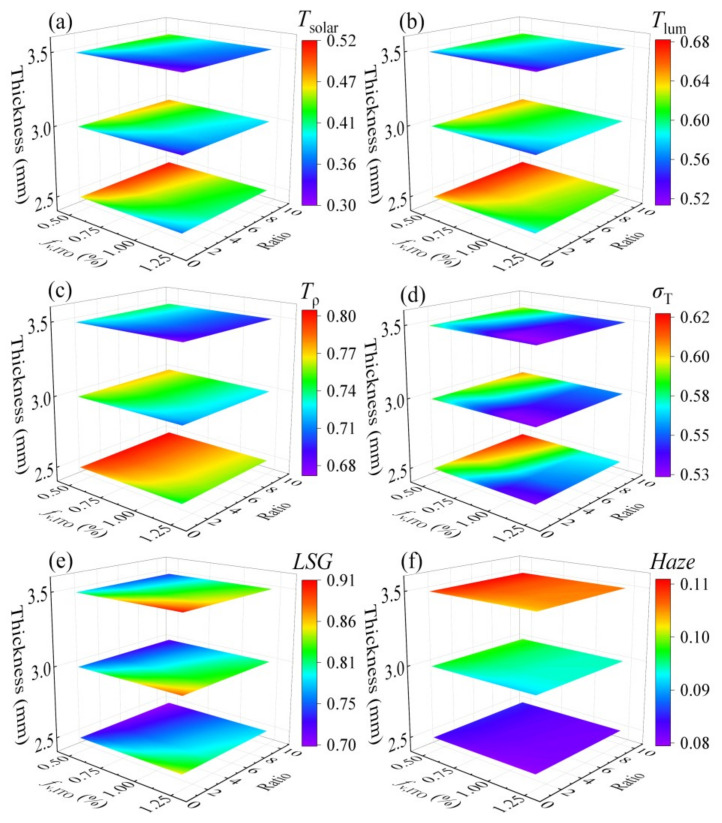
Effects of structural parameters on (**a**) solar transmittance, (**b**) visible light transmittance, (**c**) human vision efficiency, (**d**) deviation from the ideal spectrum, (**e**) light-to-solar heat gain ratio, and (**f**) haze.

Figure 6a–c show that as the aerogel thickness decreases, the transmittances (*T*_solar_, *T*_ρ_, and *T*_lum_) generally increase, reaching their maximum values at low ITO nanosphere doping concentrations and high Ag nanocylinder doping concentration ratios. Figure 6d demonstrates that as the thickness of the aerogel increases, the deviation from the ideal spectrum *σ*_T_ decreases. The configuration with ITO nanospheres at a concentration of 1.25% and Ag nanocylinders at a concentration ratio of 1 exhibits the minimal deviation from an ideal spectrum. This is partly because the inherent extinction characteristics of ITO nanospheres inevitably lead to some extinction of UV and a small fraction of visible light, contributing to this deviation when compared to a theoretical ideal glazing that transmits all visible light and blocks all non-visible light. Notably, Figure 6e reveals that the *LSG* value increases with thickness, peaking at an ITO nanosphere doping concentration of 1.25% and an Ag nanocylinder doping concentration ratio of 1. A higher *LSG* value is desirable as it indicates that, for a given amount of solar heat gain, more visible light is transmitted through the glass, signifying good light transmission efficiency relative to heat gain, and thus better insulation properties under solar load. Furthermore, haze is a critical factor in evaluating the visual experience through the glazing. As depicted in Figure 6f, the concentration of ITO nanospheres and the concentration ratio of Ag nanocylinders have a negligible impact on haze within the tested ranges. Instead, haze is primarily influenced by the thickness of the aerogel layer. Haze increases with the thickness of the spectral selective aerogel due to increased light scattering within thicker material. For a clear and unobstructed visual experience, low haze values are preferable. Despite the observed variations in haze with thickness, all three thicknesses of the spectral selective aerogels investigated in this study exhibit commendably low haze values, with the maximum not exceeding 0.12.

The ultimate goal of this optimization process is to maximize practical window performance metrics. Through comprehensive analysis, we identify an optimized configuration that achieves an exceptional balance between multiple performance parameters. Specifically, the system with 3 mm thickness, 1.25% ITO doping concentration, and concentration ratio (ratio = 1) demonstrates superior performance characteristics. This configuration simultaneously delivers high optical transparency, minimal haze effects, and effective solar modulation capability.

### 2.5. Quantifying the Real-World Impact: Building Energy Savings

**Figure 7:**
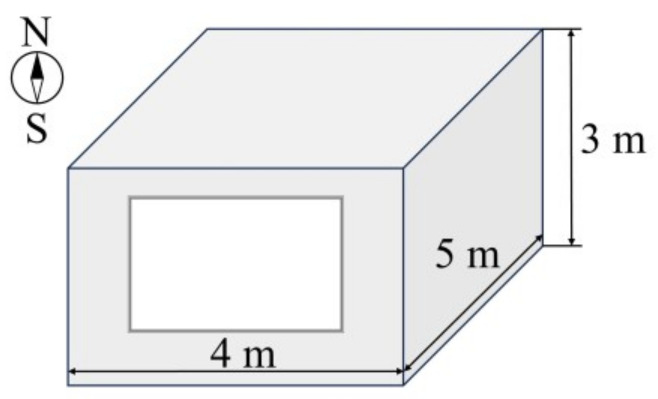
Schematic diagram of room used for simulation in TRNSYS.

**Figure 8:**
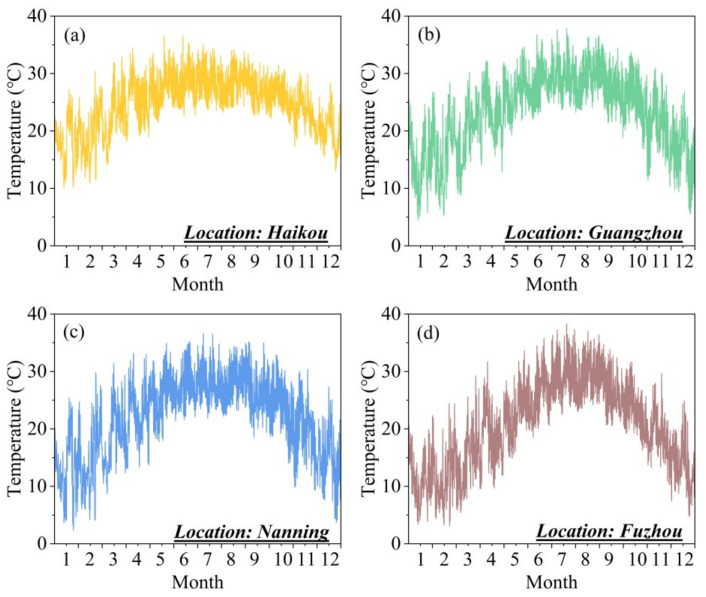
Annual average daily temperature of four locations: (**a**) Haikou, (**b**) Guangzhou, (**c**) Nanning, and (**d**) Fuzhou.

**Figure 9:**
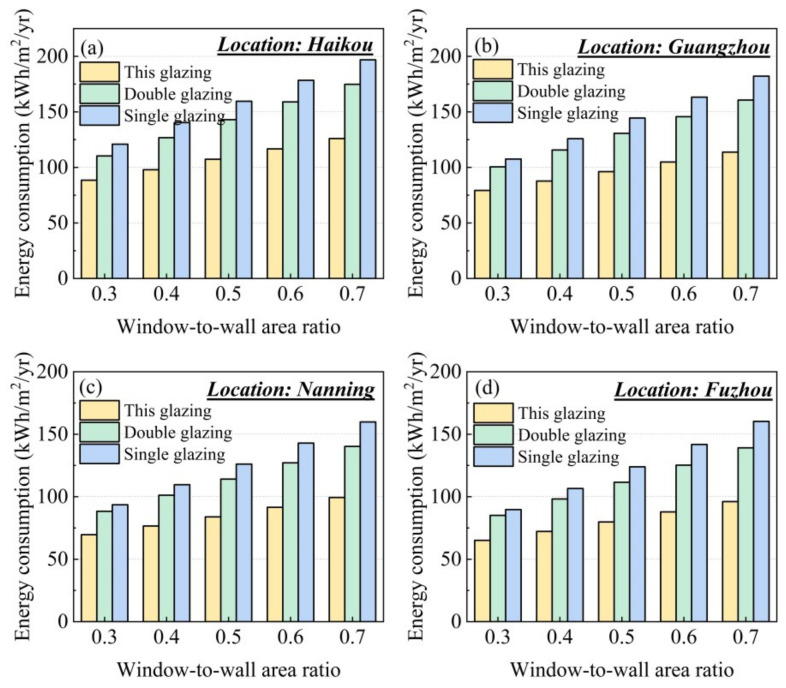
Annual energy consumption per unit area of a room with various windows and window-to-wall ratios in (**a**) Haikou, (**b**) Guangzhou, (**c**) Nanning, and (**d**) Fuzhou.

To evaluate the energy-saving performance of the nanoparticle co-doped silica aerogel window, we conducted building energy consumption simulations using TRNSYS 18, which has been discussed in detail in the literature [21]. Figure 7 illustrates our building model, which features a room dimension of 4 m × 5 m × 3 m with a south-facing window, and the window-to-wall ratio was varied from 0.3 to 0.7. We performed annual building energy consumption analyses for four representative hot-climate locations in southern China: Haikou, Guangzhou, Nanning, and Fuzhou. The geographical locations and climatic information of these locations are summarized in Table 2. The annual variations of outdoor dry-bulb temperatures for these four locations are presented in Figure 8. The meteorological data required for the simulations were obtained from the China Standard Weather Data. The results, presented in Figure 9, are unequivocal. Across all locations and all window-to-wall area ratios, the building equipped with our plasmonic aerogel window consistently demonstrates the lowest annual energy consumption, significantly outperforming both conventional single and double glazing. This superior efficiency directly stems from the aerogel’s ability to reject undesirable solar heat gain (low *SHGC*) while admitting ample daylight, a perfect combination for hot climates.

**Figure 10:**
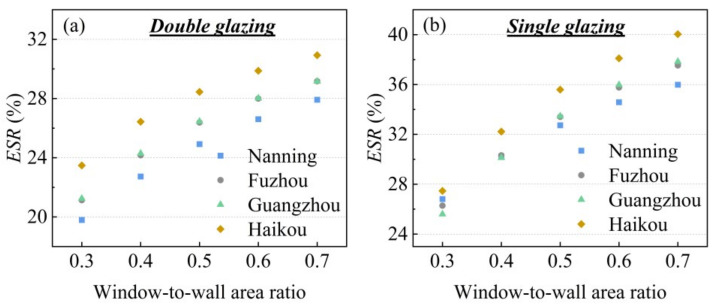
Comparison of energy saving ratios for silica aerogel-based glazing with (**a**) double glazing, (**b**) single glazing.

Figure 10 quantifies this advantage, showcasing the remarkable energy-saving ratio (*ESR*) of our window relative to the baselines. The plasmonic aerogel window achieves energy savings of 25–40% compared to single glazing and 20–31% compared to double glazing, depending on the specific climate and window-to-wall area ratio. Notably, the energy-saving benefits are most pronounced at larger window-to-wall area ratios and in the hottest climates (e.g., Haikou), where the cooling load is most severe. This outstanding performance provides the ultimate validation of our synergistic material design, proving it to be a highly effective and practical solution for enhancing building energy efficiency.

### 2.6. Economic Evaluation of Silica Aerogel Window

**Figure 11:**
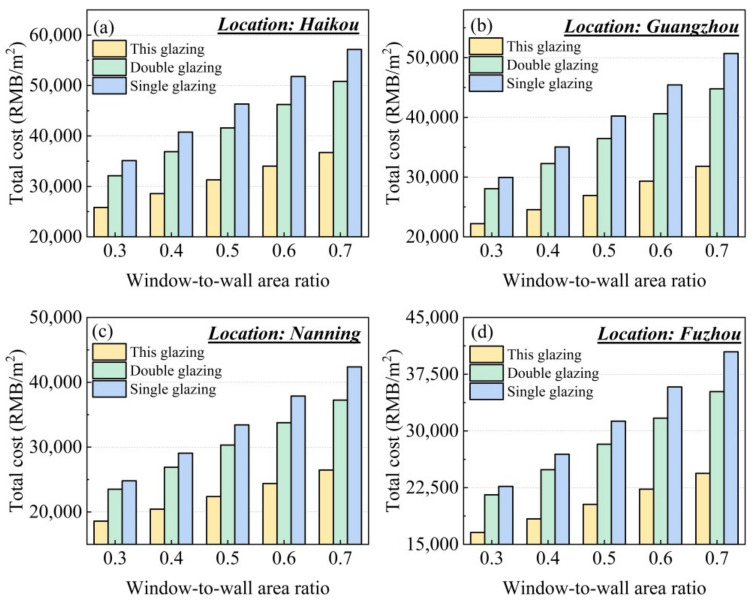
Total cost of glazing within the whole life cycle in (**a**) Haikou, (**b**) Guangzhou, (**c**) Nanning, and (**d**) Fuzhou.

The economic evaluation of nanoparticle co-doped silica aerogel windows reveals significant lifecycle cost advantages compared to conventional single-pane and double-pane windows. As shown in Figure 11, the 30-year total costs increase with higher window-to-wall ratios across all four studied cities, with Haikou and Guangzhou demonstrating particularly elevated expenses due to their hotter climates requiring substantially more cooling energy. Notably, aerogel energy-saving windows maintain consistent cost superiority in all regions, outperforming both single and double glazing by considerable margins throughout the operational period.

**Figure 12:**
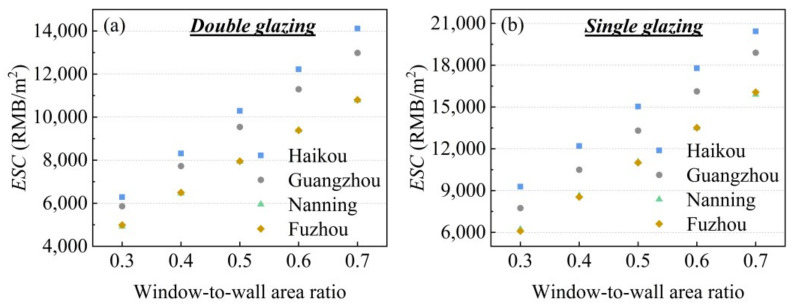
Comparison of energy cost savings for silica aerogel-based glazing with (**a**) double glazing and (**b**) single glazing.

Figure 12 demonstrates the whole life cycle energy cost savings (*ESC*) of silica aerogel windows compared to double glazing and single glazing. The results show that as the window-to-wall area ratio increases, the particle co-doped silica aerogel windows achieve cost reductions of 4987.8–14,112.6 RMB/m^2^ (equivalent to 694.3–1964.5 USD/m^2^) compared to double-glazed windows and 6096.4–20,433.6 RMB/m^2^ (equivalent to 848.6–2844.3 USD/m^2^) compared to single-glazed windows. Furthermore, the cost-saving benefits are more pronounced in Haikou and Guangzhou than in Nanning and Fuzhou.

## 3. Conclusions

In conclusion, this work has successfully designed and theoretically demonstrated a high-performance, spectrally selective silica aerogel window by pioneering a synergistic plasmonic co-doping strategy. By strategically embedding both ITO nanospheres and geometrically tuned Ag nanocylinders, we have effectively overcome the persistent trade-off between visible transparency, NIR/UV blocking, and low haze. The main conclusions are as follows:

1. Utilizing the LSPR effect, the morphology of Ag nanocylinders is adjusted to achieve precise absorption of NIR in the 0.78–0.9 μm range, while ITO nanoparticles are adjusted to block UV and a wide range of NIR. The optimized plasmonic aerogel achieves an exceptional balance of properties, including a luminous transmittance of 0.8, a haze below 0.12, and an *LSG* ratio of 0.91.
2. System-level simulations confirm the real-world impact of this design, projecting significant annual energy savings of up to 40% for buildings in hot climates when compared to conventional glazing.
3. The economic analysis demonstrates that silica aerogel energy-saving glass can achieve cost savings of up to 20,433.6 RMB/m^2^ compared to conventional glass, highlighting its significant energy efficiency benefits.

This research not only provides a practical and robust solution for energy-efficient windows but also establishes a versatile design paradigm—using synergistic, multi-component plasmonic doping—for the future development of advanced materials requiring sophisticated management of electromagnetic radiation. Furthermore, in the future, high-performance silica aerogels can be prepared using methods such as supercritical carbon dioxide drying and atmospheric pressure drying. During the sol-gel process, nanoparticles can be embedded into the internal structure, and experimental studies can be conducted on the composite mechanical properties and durability of particle-doped aerogels.

## 4. Materials and Methods

### 4.1. Conceptual Design and Physical Principles

This study proposes a novel transparent plasmonic silica aerogel, engineered for superior spectral selectivity by incorporating ITO nanospheres and Ag nanocylinders within a silica aerogel matrix. The nanoparticles are assumed to be uniformly distributed and randomly oriented throughout the substrate.

The core principle behind the enhanced optical selectivity is the excitation of LSPR in the metallic nanoparticles. When the frequency of incident solar radiation matches the natural oscillation frequency of the nanoparticles’ free electrons, LSPR occurs. This resonant phenomenon leads to a dramatic enhancement of light absorption and scattering at specific wavelengths, which are dictated by the nanoparticle’s material, size, and geometry [22]. In this work, we harness the LSPR effect of Ag nanocylinders and the intrinsic absorptivity of ITO nanospheres to selectively filter UV and NIR radiation while preserving high transmittance in the visible spectrum. The optical constants for all materials were sourced from the established literature [23].

### 4.2. Radiative Properties of Individual Nanoparticles

The radiative properties (absorption and scattering cross-sections) of the individual, non-spherical nanoparticles were calculated by solving Maxwell’s equations. While several methods exist, such as the Discrete Dipole Approximation (DDA) and Mie theory (for spheres), we selected the T-matrix method for this study [24,25,26,27]. The T-matrix method offers excellent accuracy for rotationally symmetric particles and converges significantly faster than DDA, making it more computationally efficient.

Using a validated T-matrix code, we determined the extinction (*C*_ext_), absorption (*C*_abs_), and scattering (*C*_sca_) cross-sections for individual nanoparticles based on their effective radius (*r*_eff_), aspect ratio, and complex refractive index at each wavelength. The corresponding efficiency factors (*Q*) were then calculated as follows:

(1) $$Q_{\mathrm{ext}}=\frac{C_{\mathrm{abs}}}{\pi r_{\mathrm{eff}}^{2}},$$

(2) $$Q_{\mathrm{sca}}=\frac{C_{\mathrm{sca}}}{\pi r_{\mathrm{eff}}^{2}},$$

(3) $$Q_{\mathrm{abs}}=Q_{\mathrm{ext}}-Q_{\mathrm{sca}}$$

### 4.3. Radiative Transfer in the Composite Aerogel

The radiative transfer through the nanoparticle-doped aerogel was modeled using a one-dimensional radiative transfer equation (RTE) for a homogeneous, absorbing, and scattering medium, written as follows [28,29]:

(4) $$\mu \frac{dI_{d}\left(\tau ,\mu \right)}{d\tau }=-I_{d}\left(\tau ,\mu \right)+\frac{\omega }{2}\int_{-1}^{1}P\left(\mu ,\mu^{\prime }\right)I_{d}\left(\tau ,\mu^{\prime }\right)d\mu^{\prime }+\frac{\omega }{4\pi }P\left(\mu ,\mu_{0}\right)F_{0}e^{-\tau },$$

where *μ* = cos(*θ*) is the cosine of the polar angle, *τ* = *βz* is the optical depth, ω is the single-scattering albedo, and *P* (*μ*, *μ′*) is the scattering phase function. The Henyey–Greenstein phase function was used to describe the angular distribution of scattered light [30].

(5) $$\Phi_{\mathrm{HG}}\left(\theta \right)=\frac{1}{4\pi }\frac{1-g^{2}}{{\left(1+g^{2}-2g\cos\theta \right)}^{3/2}},$$

The RTE was solved using a robust Monte Carlo (MC) simulation code, which has been extensively validated in our previous works [31,32]. The key inputs for the MC model are the spectral bulk optical properties of the composite material, which were calculated as follows, assuming independent scattering.

Absorption and scattering coefficients (*β*_abs_, *β*_sca_): The total coefficients of the composite are the sum of contributions from the silica aerogel matrix (m) and the doped nanoparticles (p) [33]. The nanoparticle contributions are determined by their efficiency factors *Q* and volume fraction (*f*_v_), written as follows [33,34]:

(6) $$\beta_{\mathrm{abs}}=\beta_{\mathrm{abs},p}+\beta_{\mathrm{abs},m},$$

(7) $$\beta_{\mathrm{sca}}=\beta_{\mathrm{sca},p}+\beta_{\mathrm{sca},m},$$

(8) $$\beta_{\mathrm{abs},p}=\frac{3}{4}Q_{\mathrm{abs}}\frac{f_{v}}{r_{\mathrm{eff}}},$$

(9) $$\beta_{\mathrm{sca},p}=\frac{3}{4}Q_{\mathrm{sca}}\frac{f_{v}}{r_{\mathrm{eff}}}$$

Effective refractive index (*n*_eff_): The effective refractive index of the composite medium was calculated using the Maxwell–Garnett effective medium theory (EMT), written as follows [35]:

(10) $$\frac{n_{\mathrm{eff}}-n_{m}}{n_{\mathrm{eff}}+2n_{m}}=\sum_{i=1}^{j}f_{v,i}\frac{n_{i}-n_{m}}{n_{i}+2n_{m}}$$

### 4.4. Window Performance Metrics

Based on the spectral transmittance (*T*_λ_), reflectance (*R*_λ_), and absorptance (*A*_λ_) obtained from the MC simulations, we evaluated the overall window performance using the following standard metrics:

Solar transmittance (*T*_sol_) is the fraction of total solar energy transmitted through the glazing, weighted by the AM1.5 solar spectrum (*I*_AM1.5_) [36].

(11) $$T_{\mathrm{sol}}=\frac{\int_{0.3\;\mu m}^{2.5\;\mu m}T_{\lambda }I_{\mathrm{AM}1.5}\left(\lambda \right)d\lambda }{\int_{0.3\;\mu m}^{2.5\;\mu m}I_{\mathrm{AM}1.5}\left(\lambda \right)d\lambda }$$

Visible light transmittance (*T*_ρ_) is the fraction of visible light transmitted through the glazing, weighted by the AM1.5 solar spectrum (*I*_AM1.5_) [36].

(12) $$T_{\rho }=\frac{\int_{0.38\;\mu m}^{0.78\;\mu m}T_{\lambda }I_{\mathrm{AM}1.5}\left(\lambda \right)d\lambda }{\int_{0.38\;\mu m}^{0.78\;\mu m}I_{\mathrm{AM}1.5}\left(\lambda \right)d\lambda }$$

Luminous transmittance (*T*_lum_) is the fraction of visible light transmitted, weighted by the standard luminous efficiency function of the human eye, *V*(*λ*), to represent perceived brightness [37,38].

(13) $$T_{\mathrm{lum}}=\frac{\int_{0.38\;\mu m}^{0.78\;\mu m}T_{\lambda }V\left(\lambda \right)d\lambda }{\int_{0.38\;\mu m}^{0.78\;\mu m}V\left(\lambda \right)d\lambda }$$

Haze represents the percentage of transmitted light that is scattered forward by more than a specified angle (5° conical angle), quantifying the optical clarity. It is calculated as the ratio of diffuse transmittance to total transmittance [18].

(14) $$Haze=\frac{T_{\mathrm{nh}}-T_{\mathrm{nn}}}{T_{\mathrm{nh}}}$$

Light-to-solar heat gain ratio is defined as the ratio of luminous transmittance to the solar heat gain coefficient (*SHGC*) [39]. A higher *LSG* indicates better spectral selectivity.

(15) $$LSG=\frac{T_{\mathrm{lum}}}{SHGC}$$

Deviation from ideal spectrum (*σ*_T_) is a metric to quantify how closely the glazing’s spectral absorptance (*A*_λ_) matches an ideal spectrum (*A*_i_), which absorbs all UV and NIR light but transmits all visible light [40].

(16) $$\sigma_{T}=\frac{\int_{0.3\;\mu m}^{2.5\;\mu m}\sqrt{\left(A_{i}-A_{\lambda }\right)}I_{\mathrm{AM}1.5}\left(\lambda \right)d\lambda }{\int_{0.3\;\mu m}^{2.5\;\mu m}A_{\lambda }I_{\mathrm{AM}1.5}\left(\lambda \right)d\lambda }$$

(17) A=1−T−R

### 4.5. Building Energy Consumption Simulations

Annual building energy consumption simulations were performed using the TRNSYS 18 software. The performance of our optimized plasmonic aerogel glazing was benchmarked against conventional single glazing and double glazing. Key thermal and optical properties for all window types used as inputs for TRNSYS are summarized in Table 3. The total annual energy consumption (*E*), including both cooling and heating loads, and the energy-saving ratio (*ESR*) were calculated as follows:

(18) $$E=E_{\mathrm{cool}}+E_{\mathrm{heat}},$$

(19) $$ESR=\frac{E_{\mathrm{base}}-E}{E_{\mathrm{base}}}\times 100\%$$

where *E*_base_ is the energy consumption of the building with the baseline window (single or double glazing) and *E* is the consumption with our plasmonic aerogel window.

### 4.6. Economic Evaluation Method

The lifecycle economic evaluation of silica aerogel windows incorporates both initial costs and operational costs. For simplified analysis, this study assumes negligible initial costs associated with installation and maintenance, while operational costs primarily consist of electricity consumption required to maintain indoor thermal comfort. Regional electricity pricing data are presented in Table 4, and comparative costs of different window types are detailed in Table 5, where the cost of silica aerogel windows is based on the expected cost of future large-scale production. This forward-looking assumption is common in evaluating emerging technologies to assess their long-term viability. The total cost (*TC*) of the entire life cycle process is expressed as follows [41,42]:

(20) TC=IC+EC⋅PWF

where *IC* is the initial cost of windows, *EC* represents the energy cost, and *PWF* is the present worth factor. *EC* can be calculated by

(21) $$EC=\frac{E}{COP}c_{\mathrm{elec}}$$

where *E* is the total building energy consumption, *c*_elec_ is the price of electricity and *COP* is the coefficient of performance of the air conditioning system, assumed to be 3.

The present worth factor *PWF* is the coefficient that converts the total energy cost over the whole life cycle into direct costs and is calculated by the following [43]:

(22) $$PWF=\frac{1}{i-r}\left[1-{\left(\frac{1+r}{1+i}\right)}^{N}\right]$$

where *i* is the bank interest rate, set at 2%, *r* is the inflation rate, assumed to be 2.5%, and *N* represents the life cycle, estimated to be 30 years. Therefore, compared to the base window, the whole life cycle energy cost savings *ESC* of silica aerogel window can be calculated by the following [44]:

(23) $$ESC=TC_{\mathrm{base}}-TC_{\mathrm{aerogel}}$$

## Figures and Tables

**Table 1:** Doping concentration ratio of Ag nanocylinders with aspect ratios of 0.13 and 0.15.

*f*_v,AR = 1.3_	*f*_v,AR = 1.5_	*Ratio*
1 × 10^−6^	1 × 10^−7^	10
1 × 10^−6^	2.5 × 10^−7^	4
1 × 10^−6^	5 × 10^−7^	2
1 × 10^−6^	7.5 × 10^−7^	1.33
1 × 10^−6^	1 × 10^−6^	1

**Table 2:** Basic information of cities in this work.

Locations	Longitude and Latitude	Altitude	Climate
Haikou	20° N, 110°15′ E	63.5 m	Tropical maritime monsoon climate
Guangzhou	23°13′ N, 113°29′ E	70.7 m	Subtropical maritime monsoon climate
Nanning	22°38′ N, 108°13′ E	121.6 m	Subtropical monsoon climate
Fuzhou	26°05′ N, 119°17′ E	84 m	Subtropical monsoon climate

**Table 3:** *U*-value, *SHGC*, and *T*_sol_ of the silica aerogel-based energy-saving glass.

	*Thickness* (mm)	*U* (W/m^2^K)	*SHGC*	*T*_sol_
This glazing	3	3.09	0.547	0.476
Double glazing	6	2.97	0.64	0.607
Single glazing	6	5.41	0.823	0.791

**Table 4:** Electricity price in different locations in China.

Location	Electricity Price (RMB/kWh)
Haikou	0.9183
Guangzhou	0.8802
Nanning	0.8383
Fuzhou	0.7983

**Table 5:** Comparative cost analysis of window types.

Window Type	Initial Cost (RMB/m^2^)
Silica aerogel	200
Double glazing	102
Single glazing	30

## Data Availability

Data underlying the results presented in this paper are not publicly available at this time but may be obtained from the authors upon reasonable request.

## Abbreviations

The following abbreviations are used in this manuscript:

*A*	absorptance
*C*	cross-section
DDA	discrete dipole approximation
*E*	energy consumption
*ESR*	energy saving ratio
*f*_v_	particle volume fraction
*F*_0_	incident flux
*g*	asymmetry factor
*I*_d_(*τ*,*μ*)	the diffuse intensity
*I*_AM1.5_(*λ*)	solar irradiation intensity
*LSG*	light to solar heat gain ratio
MC	the Monte Carlo method
*n*	refractive index
*Q*	efficiency factor
*r*_eff_	effective radius of a cluster of monodispersed spheres, nm
*T*	transmittance
*V*(*λ*)	standard luminous efficiency function
*β*	coefficient, m^−1^
*θ*	polar angle, °
*λ*	wavelength, μm
*μ*	cosine of the polar angle
*ρ*	visible light
*σ*_T_	deviation from the ideal spectrum
*τ*	optical coordinat
*ω*	the single scattering albedo
abs	absorption
base	base window
cool	cooling
eff	effective
heat	heating
lum	luminous
m	medium
p	particle
sca	scattering
sol	solar
